# Supplementary material for: Causal interplay between lactose intolerance and gut microbiota: a combined bidirectional Mendelian randomization and in vivo validation study
Source: Front Nutr. 2026 Jun 1;13:1803337. doi: 10.3389/fnut.2026.1803337 (PMC13265576; doi:10.3389/fnut.2026.1803337)
Supplement: Supplementary file 1 [file Data_Sheet_1.zip › supplementary materials/Forward/leave one out cross validation/ebi-a-GCST90027508.finngen_R12_E4_LACTONAS.pdf]

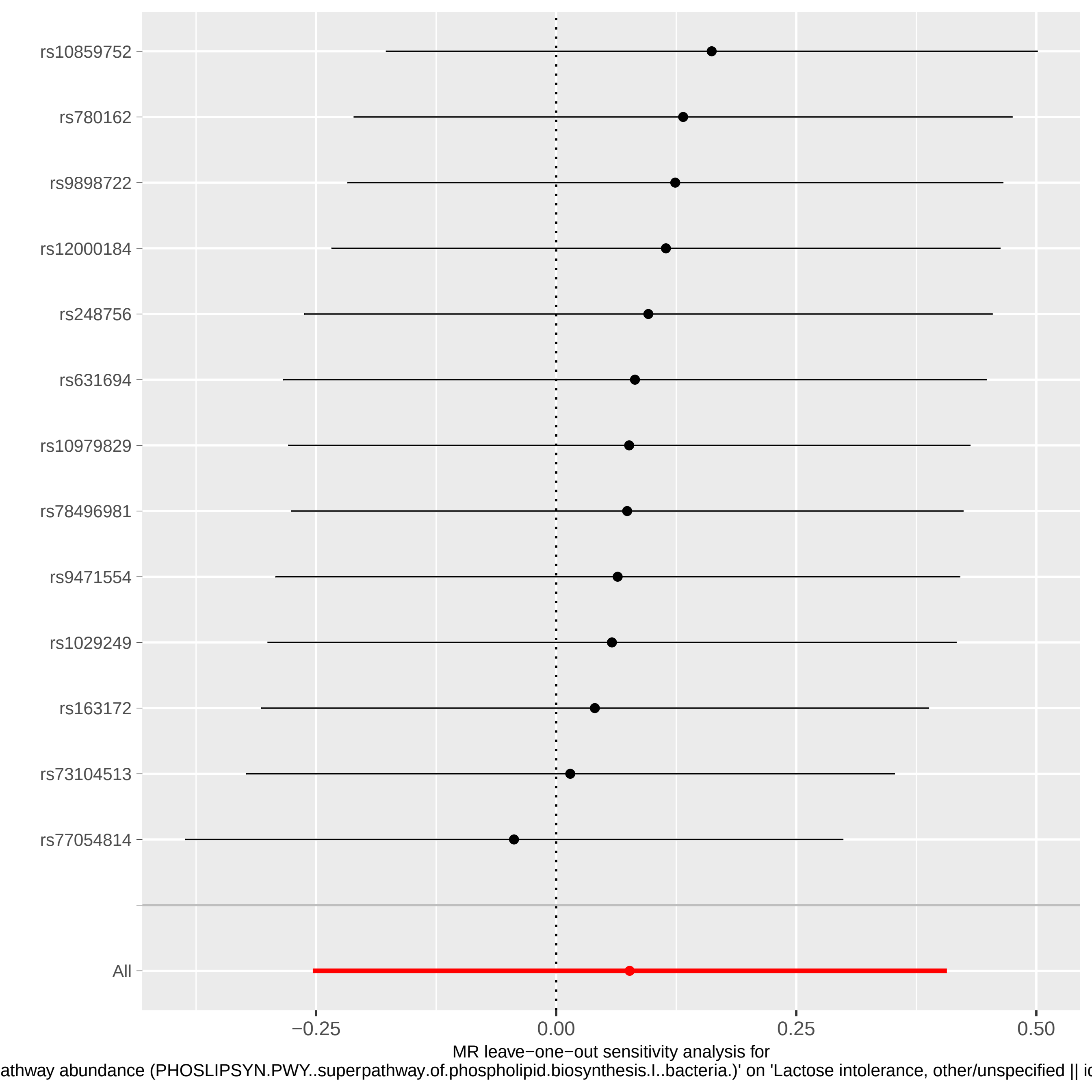

rs10859752
rs780162
rs9898722
rs12000184
rs248756
rs631694
rs10979829
rs78496981
rs9471554
rs1029249
rs163172
rs73104513
rs77054814
All
−0.25
0.00
0.25
0.50
MR leave−one−out sensitivity analysis for
athway abundance (PHOSLIPSYN.PWY..superpathway.of.phospholipid.biosynthesis.I..bacteria.)' on 'Lactose intolerance, other/unspecified || ic
